# Evidence-based sentencing and scientific evidence

**DOI:** 10.3389/fpsyg.2023.1309141

**Published:** 2023-11-14

**Authors:** Lucía Martínez-Garay

**Affiliations:** Departament de Dret Penal, Universitat de València, Valencia, Spain

**Keywords:** evidence-based sentencing, violence risk assessments, evidence-based policies, scientific evidence, criminal law, selective incapacitation, rehabilitation

## Abstract

Evidence-based sentencing (EBS) is a new name for an aspiration that has deep roots in criminal law: to apply the sentence most appropriate to each offender's risk of reoffending, in order to reduce that risk as far as possible. This modern version of the traditional sentencing goals of rehabilitation and incapacitation fits into the broader approach of so-called “evidence-based public policy.” It takes the view that the best existing evidence for reducing reoffending are modern structured risk assessment tools and claims to be able to achieve several goals at once: reducing reoffending, maintaining high levels of public safety, making more efficient use of public resources, and moving criminal policy away from ideological battles by basing it on the objective knowledge provided by the best available scientific evidence. However, despite the success of this approach in recent years, it is not clear to what extent it succeeds in correctly assessing the risk of individual offenders, nor whether it achieves its intended effect of reducing recidivism. This paper aims to critically examine these two issues: the quality of the scientific evidence on which EBS is based, and the available data on the extent to which it achieves (or does not achieve) its intended goals.

## 1. Introduction

The evidence provided by social science on the effects of punishment has been systematically ignored by US criminal justice policy for at least 40 years.^1^ The second half of the 1980s and the whole of the 1990s were marked by “tough on crime” and the “war on drugs”: the number of crimes punishable by life without parole increased, parole was abolished in many states and at the federal level, harsh mandatory minimum sentencing laws were enacted, three-strikes laws were introduced, harsher sentences or civil commitment were introduced for sex offenders, and punishments were generally increased, including for juveniles. The results of these criminal policies are well known: incarceration rates soared to a peak of 2,310,300 people in prison in 2008, a rate of 760 per 100,000 residents,^2^ with huge racial disparities and at a cost of billions to the federal and states governments, among other problems.^3^

None of these initiatives had evidence that they were effective in reducing crime (Haggerty, 2004, p. 220; Tonry, 2013b). These policies were apparently intended to reduce crime through the deterrent and incapacitating effects of harsher sanctions^4^ (Tonry, 2013b, p. 159), but the studies available at the time, and also those that have been published subsequently, showed that such effects are minimal, non-existent, or even counterproductive (cf. Tonry, 2013b, p. 175 ff.; National Research Council, 2014, p. 337, 339).

Since the late 1990s, however, and in a context in which US crime policy has moved away from the more extreme forms of “tough on crime,” the popularity of what has come to be known as “evidence-based practices” has been growing. This term encompasses a range of techniques and strategies used by courts, correctional institutions and community supervision agencies, that aim to tailor criminal justice interventions to the characteristics of different individuals and groups in order to make them more effective (Klingele, 2015, p. 539). More specifically, these are “policies, procedures and programs that scientific research has demonstrated to reduce recidivism for specific offender populations such as probationers, parolees, and drug-addicted offenders” (National Conference of State Legislatures, 2011). The scientific evidence referred to in these approaches is the accumulated knowledge from criminology and psychology about the risk factors associated with an increased likelihood of violent or criminal behavior, and the existing knowledge about the most effective intervention programmes and techniques to reduce this risk in specific groups of offenders.

Against the background we have summarized in the previous lines, the name given to these practices, “evidence-based,” is immediately striking: could it be that after more than 40 years of disregard for scientific evidence we are witnessing a change in trend, and that American criminal policy about criminalization, imposition and execution of sentences is really being designed according to the scientific knowledge that the social sciences have been accumulating on the effects of sentences? I fear that, unfortunately, this may not be the case, and that the appeal to “scientific evidence,” although it is justified in some cases, runs the risk of becoming a means of legitimizing practices that have not been shown to be effective in reducing crime, and of presenting political choices as if they were mere technical issues.

## 2. What is evidence-based sentencing

The field of crime control covers many areas in which evidence-based practices can be applied: police investigations and arrests (evidence-based policing), the pre-trial phase and the adoption of precautionary measures, the sentencing phase, and the enforcement of the penalties (evidence-based corrections).

In the evidence-based sentencing approach (hereinafter EBS) there is a very close link between the moment when the sentence is imposed and its subsequent enforcement, since the aim is to impose sentences that, due to their characteristics, allow the implementation of the programmes that are considered most appropriate to reduce the risk of reoffending (Casey et al., 2011, p. 7). EBS includes decisions related to the granting of probation, its possible revocation and the supervision/treatment regime that has to accompany it. In the words of one author who has strongly promoted the adoption of this perspective, EBS consists of “sentencing and corrections policies and programs based on the best research evidence of practices shown to be effective in reducing recidivism” (Warren, 2010, p. 153).^5^

Although I will use the term evidence-based sentencing, the terminology is quite variable. It is sometimes referred to as “risk-based sentencing” (Slobogin, 2019); “effective sentencing” (National Conference of State Legislatures, 2011), “actuarial sentencing” (Hannah-Mofatt, 2013), “smart sentencing” (Marcus, 2006) or “predictive sentencing” (De Keijser et al., 2019). All these denominations refer to essentially the same thing, although each one emphasizes one of the different characteristics of the phenomenon. Indeed, the scientific evidence on which EBS is based are the actuarial or structured tools for assessing the risk of recidivism (or of specific risks, like violent or sexual recidivism), which have been developed in criminology in recent decades (actuarial sentencing)^6^; these tools make it possible to estimate the level of risk of each offender, i.e., to predict the likelihood of future criminal behavior (predictive sentencing); what EBS proposes is to use this level of risk as the central criterion for deciding the most appropriate sentence for the subject (risk-based sentencing), claiming that this will allow for a more efficient use of public resources (effective sentencing) by concentrating them on the subjects who really need them, and avoiding long sentences and intensive interventions on those at low risk. All of this means, it is said, a much more intelligent management of the criminal problem (smart sentencing), in so far as with the same or fewer public resources, it will be possible to reduce recidivism and better protect the community.^7^

The success of this approach in the US is remarkable, as it is being implemented—to a greater or lesser extent—in many of its jurisdictions.^8^ However, it is also generating an intense debate, as the use of risk assessment tools in criminal law (and not only in sentencing) raises important problems and challenges.^9^ In this paper I will address only one of them: the quality of the scientific evidence on which EBS is based, and what empirical evidence there is that it achieves its intended objectives. Before undertaking this analysis, however, it is worth offering a brief overview of the context (historical, political and epistemological) in which evidence-based sentencing has emerged.

### 2.1. The principles of effective correctional intervention and the “Risk-Need-Responsivity” model

In the field of criminal justice, the first so-called “evidence-based” programmes and procedures emerged in the field of corrections, following the discrediting of the rehabilitative ideal since the 1980s. Faced with the hostile environment created by the then dominant “tough-on-crime” policies, professionals who still believed in rehabilitation, in the criminogenic effects of prison and in the possibility of improvement for many offenders, sought to gather evidence that some rehabilitation programmes and interventions did work. Thus, debate ensued about which treatment programmes were effective or more effective, and—based on the work of Andrews and Bonta among others—a theoretical model was built to systematize and explain the “principles of effective correctional intervention,” which crystallized in the model known as “Risk, Need and Responsivity,” or RNR (Velásquez Valenzuela, 2014, p. 70 ff., Klingele, 2015, p. 552 f.). This model is built around the central notion of risk, and is based—very briefly—on the following assumptions: (1) that the risk of recidivism is measurable and that this can be done today with structured or actuarial methods much more reliably than with the traditional clinical method; (2) that interventions should be tailored to each individual's level of risk (intensive interventions are not advisable for low-risk individuals, as they may even have counterproductive effects, and that efforts and resources should be concentrated on higher-risk individuals); (3) that each subject has specific needs (medical, economic, cognitive-behavioral, educational, etc.) that favor the commission of new crimes, and that therefore acting on these needs can reduce the level of risk; and (4) that interventions must be adjusted to the receptiveness of each subject or group of subjects, as not everyone responds in the same way to the same programmes or techniques (Andrews and Bonta, 2010, p. 44 et seq.).

These studies managed to build a “theory on effective rehabilitation” (Velásquez Valenzuela, 2014, p. 78), which also managed to carve out an important niche in the criminal policy debate from the 1990s onwards. However, probably the part of this approach that is proving most decisive in current criminal policy is not that of rehabilitation, but that of effectiveness, as we will see below.

### 2.2. Managerial-actuarial justice and effective risk management

The development of the principles of effective intervention described in the previous section was parallel in time to another line of evolution in American criminal policy: the progressive incorporation of criminal risk management techniques based on the managerial-actuarial model. In recent decades, a new rationality has emerged in crime control, characterized by an approach based on the efficient management of the system's resources. From this perspective, crime is seen not so much as a serious problem to be eliminated or fought, but as a phenomenon inherent to any society, which can be managed with greater or lesser efficiency, taking into account the resources available. For this perspective, risk is also a central concept, although not exactly in the same sense as it is seen in the paradigm of the RNR model referred to in the previous section. The managerial-actuarial model is little concerned with the causes of crime and does not see rehabilitation as a main objective; rehabilitative programmes may be used if they are cost-effective, but the central aim is to keep the level of deviance under control within tolerable margins. Risk level indicators measured with actuarial instruments are used for this purpose because they are useful to manage large populations in an objective way and with a minimum of costs. The central concern for this approach is resource efficiency and cost optimization (Velásquez Valenzuela, 2014; Klingele, 2015, p. 545 ff, 572 ff; Brandariz García, 2016, p. 93 ff, 111 ff; Castro Liñares, 2019, p. 79 ff).

It is in this context that the relative success of evidence-based rehabilitation programmes must be placed. As Klingele (2015, p. 552) highlights, practitioners and criminologists seeking to persuade managers and politicians reluctant to allocate financial resources to rehabilitation and treatment programmes sought to demonstrate that they were worth investing in because they produced measurable benefits in terms of crime reduction by accurately documenting outcomes and gathering objective data to support their effectiveness in reducing reoffending.^10^ The emphasis on cost-effectiveness was intended to provide funding and opportunities for programmes that could not be pursued by appealing only to the intrinsic value of the rehabilitative ideal as a legitimate and just objective of criminal sanctions.

The goal has been achieved, at least in part. Today, the old aphorism that “nothing works”^11^ is considered to have been overcome and it is accepted that certain programmes and treatments have been shown to be effective in reducing reoffending, especially those based on the RNR model, and that this knowledge should be used by the administration of justice; and this is a fundamental premise of all the documents produced by official bodies, professional associations, think tanks or other interest groups related to the administration of justice that promote the use of EBS. However, what has been incorporated into the official discourse of EBS is the possibility of *effectively* reducing recidivism, but not so much the fact that this reduction must be achieved through the *rehabilitation* of convicted offenders. This is easy to see if one examines the objectives which, according to these documents, EBS should pursue: the main objective proclaimed in all of them is the reduction of recidivism, always accompanied by a reference to the improvement of public safety and the more effective use of public resources; on the other hand, references to the rehabilitation of convicted offenders, if they are present at all, occupy a very vague second place.^12^ Nor should the objective of reducing the prison population, which is also found in almost all initiatives supporting the EBS,^13^ be confused with rehabilitation: the main reason for reducing the prison population is that it represents a very high cost for the administration, without mass imprisonment having proved to be an effective means of reducing crime. It is true that EBS seeks to avoid the criminogenic effects of imprisonment, but the main reason for reducing the prison population is to save costs,^14^ and do so by means that have been shown to be as effective as imprisonment in protecting public safety (and therefore more effective in terms of cost-benefit).^15^

“Effective rehabilitation,” as conceived by Andrews and Bonta and others, has undoubtedly been a fundamental pillar for the maintenance and expansion of programmes aimed at the rehabilitation of convicts, both inside and outside prison, but in the EBS approach the dimension of effectiveness prevails over that of rehabilitation, and violence risk assessments are primarily at the service of an effective control of the convicted population in terms of cost-benefit. If rehabilitation programmes are the most effective option for achieving this goal, resources will be allocated to them, but when this is not the case, control will be exercised through other mechanisms.

### 2.3. Evidence-based policies

Before critically examining the quality of the scientific evidence on which evidence-based sentencing is based, it is worth alluding, albeit as briefly as space permits, to the choice of the term by which this trend is called: by incorporating the term “evidence-based,” EBS does not refer to any way in which the design, imposition and enforcement of sentences could be supported by the knowledge that the social science provides about the effects of criminal sanctions, but deliberately inserts itself into a certain, much more general movement known as evidence-based policy or evidence-based policymaking.^16^ This approach builds on the success and prestige of evidence-based medicine in the early 1990s and considers, very succinctly, that: (1) there is a hierarchy of quality of scientific evidence, with randomized control trials and meta-analyses at the top, and the practitioner's clinical experience at the bottom; (2) not only in medicine, but also in the design of all kinds of public policies, decisions must be based on the best available scientific evidence, understood according to the hierarchy just mentioned; and (3) decisions based on this kind of scientific evidence are better because, on the one hand, they have been proven to be effective in achieving the proposed goal and, on the other hand, because they are no longer (or not only) motivated by political objectives—in the sense of ideological or partisan interests—but are based on objective data on what works and what does not in solving problems. In this approach, being “evidence-based” is seen as a necessary condition for greater transparency, accountability and better governance (Strassheim and Kettunen, 2014, p. 259), which ultimately adds legitimacy.^17^

There is no doubt that the idea of developing evidence-based policy is immediately attractive,^18^ including for criminal law,^19^ and the evidence-based policymaking movement has considerable support,^20^ with numerous governmental and non-governmental agencies now seeking to increase the use of scientific evidence in the design of public policy (Parkhurst, 2016, p. 16).

However, the actual implementation of evidence-based policies is much more complex than it appears, and the evidence-based policy approach has been heavily criticized.^21^ It has been argued that it is based on a linear understanding of the relationship between scientific knowledge and policy practice, according to which science would be able to identify “the best” solution to any social problem, which, once identified, would simply have to be implemented (Greenhalgh and Russell, 2009, p. 305). However, the scientific evidence on a given issue is often not unambiguous; moreover, the timescales of politics and science are very different, and in political decisions there are other factors in addition to scientific knowledge that legitimately condition decisions (Klein, 2000; Parkhurst, 2016). On the other hand, evidence-based policy would reflect a positivist understanding of scientific knowledge according to which social problems are “out there,” waiting for someone to identify them and provide a solution, whereas in fact the identification of something as a problem, its definition, and the priority given to it in the political agenda depend on a social construction in which the competing values and interests of different groups and different ideologies are juxtaposed (Greenhalgh and Russell, 2009, p. 315).^22^ The emphasis by proponents of evidence-based policymaking on the need to adopt “what works” policies, which tends to present problems as purely technical issues, is misleading, because it obscures the political nature of many of the problems to be solved: the fact that there is a solution for something for which we have scientific evidence of effectiveness does not mean that it is an important problem to solve; scientific research can provide evidence about what the consequences of particular policies are, but it cannot alone determine which of those policies should be considered preferable.^23^

Evidence-based policymaking also privileges a certain type of scientific evidence (randomized trials, quantitative measurements, statistical methods), whereas in the social sciences there are many other types of analysis that can provide relevant knowledge about the complexity of the relationships and tensions inherent in social reality.^24^ In fact, most social phenomena cannot be measured with the precision with which, for example, the effect of a certain antibiotic on the number of bacteria present in an organism is measured; if, in this situation, the “evidence” to be taken into account in the design of a policy excludes that which does not come from the sources previously established as preferential, there is a monopolization of the knowledge considered relevant and an oversimplification of reality (Strassheim and Kettunen, 2014, p. 263). As Saltelli and Giampietro state: “Once the analysis has removed all sources of uncomfortable knowledge the problem reduces to one which can be treated by the usual combination of cost benefit analysis and risk analyses methodologies, and the solution optimized to the desired precision, be it that the solution may have lost at this stage all its relevance to the original problem” (Saltelli and Giampietro, 2017, p. 66). In addition, in the not uncommon situation where there are scientific studies with conflicting results on a given issue, the decision-maker or policy-maker may deliberately select only the scientific evidence that supports the decision already taken on the basis of other criteria and interests (cherry picking).

As a result of all these problems, other approaches have also emerged that broaden the type of scientific evidence considered relevant for evaluating public policies in relation to complex social problems, moving away from an exclusive focus on randomized control trials and meta-analysis, and combining qualitative and quantitative methods in a flexible way. One example is realist evaluation, developed by Pawson and Tilley (1997), which recognizes that programmes may work for some people in some circumstances, but not for others in different contexts. They should therefore not be judged from an all-or-nothing (it works/it does not) perspective that focuses only on the level of programme effectiveness, but evaluations should look at the underlying causal mechanisms that explain different outcomes for different groups, and also at unintended consequences (Pawson et al., 2005; Croci et al., 2023).

In short, the (laudable) goal of designing and implementing policies that are more transparent and effective because they are based on solid knowledge of their effects and consequences is by no means easy to achieve, as it is threatened by at least two risks: on the one hand, the risk of passing off as “evidence-based policy” what in reality is nothing more than “policy-based evidence,” i.e., the selective use of data to legitimize pre-established policy objectives with this supposed scientific evidence (Strassheim and Kettunen, 2014, p. 262; Parkhurst, 2016, p. 48 f.); and, on the other hand, there is also the danger of crowding out open and legitimate debate on competing ideological and moral options by hiding the political dimension of the arguments at stake behind the apparent neutrality of better or more consistent scientific support for one of the options in dispute.

Against this background, it is interesting to analyse the extent to which evidence-based sentencing really incorporates “the best available evidence” on crime control, and whether it is scientifically proven that designing criminal sanctions on the basis of recidivism risk estimates is a policy “that works.”

## 3. Evidence-based sentencing and scientific evidence

### 3.1. The quality of evidence on which evidence-based sentencing is based

The scientific evidence on which EBS is based consists mainly of structured assessments of the risk of reoffending.^25^ And probably the majority view is that such estimates provide robust and verified information about the likelihood of recidivism or violent reoffending. It is repeatedly claimed that modern risk estimates, made using structured methods (whether these are purely actuarial or structured clinical judgement),^26^ are far more reliable than those made using purely clinical judgement, and that accuracy rates are higher today than they were 30 or 40 years ago.^27^ However, while there have certainly been important improvements in the understanding of violence risk assessment since the 1980s, to simply say that the predictive accuracy of modern structured instruments is better than the clinical judgement of practitioners oversimplifies the issue, and thus paints an overly optimistic and potentially misleading picture.

To put the problem in perspective, it is worth recalling that what contributed decisively to the discrediting of the old clinical assessments of dangerousness in the 1970s and 1980s was the realization that they produced a certain type of error, false positives, in proportions that were considered excessive at the time. Studies showed that, out of every three people considered dangerous by a psychiatrist or psychologist, only one actually went on to commit violent acts (Monahan, 1981, p. 77). The fact that two out of three predictions of violent behavior were disproved by the subsequent behavior of the subject was considered to be evidence of insufficient quality on which to base major restrictions of rights, such as the ordering or prolongation of psychiatric or penal detention.

Today, however, this type of error has not been significantly reduced. The statistical indicator that measures the percentage of subjects who actually reoffend, out of the set of those who have been assessed as high risk, is called the positive predictive value. In the studies on violent reoffending conducted internationally, this value is usually below 50%, and often significantly lower (Douglas et al., 2017). In a relevant and well-known meta-analysis that examined the use of nine risk assessment tools in 73 studies involving more than 24,000 subjects, the positive predictive value for assessing the risk of violent recidivism was on average 41%, i.e., for every 10 subjects who were considered to be at high risk, only 4 committed further violent acts. In the same study, the positive predictive value of tools used to estimate the risk of sexual recidivism was on average 23% (Fazel et al., 2012, p. 10). More recently published studies continue to show positive predictive values for the likelihood of violent reoffending that do not even reach 40%.^28^

In the light of these data, it does appear that risk assessments have improved much in terms of predictive accuracy over the clinical judgements of 40 years ago.^29^ So where does the widespread belief come from that modern structured risk assessments are much better than clinical judgements? In my view, there are two main reasons: the parameters by which a risk assessment is judged to be good have changed, and the way in which information about the quality of the estimates is communicated has also changed.

Regarding the former, decades ago predictions of dangerousness were considered binary, in the sense that both the future event and the prediction had only two possible outcomes: the new offense either occurred or it did not; and the practitioner could either have considered the subject to be dangerous or not. In this type of prediction, there are only four possible outcomes: true negative (considered not dangerous and did not reoffend), false negative (considered not dangerous but did reoffend), true positive (considered dangerous and did reoffend) and false positive (considered dangerous but did not reoffend).

Since the 1990s, however, a distinction has been made between the occurrence or non-occurrence of the event, and the different degrees of confidence one can have that the event will occur (Mossman, 2006, p. 549 et seq., 555 et seq.). Although reoffending is a binary event (either the assessed person commits new offenses, or they do not^30^), the judgement one makes about the likelihood of its occurrence is not, because the assessor may consider that level to be not only high or low, but also very low, low, medium, high, very high, extreme, and so on. If, for example, a risk assessment tool classifies a group of people with a subsequent reoffending rate of 30% as high risk, a group with a reoffending rate of 20% as medium risk, and others with a reoffending rate of only 10% as low risk, it seems clear that it can distinguish which groups of people are at higher risk than others. In this sense, it can be said that it works well, or that its predictive accuracy is “good,” at least in terms of its ability to discriminate the greater or lesser relative risk of reoffending of some groups compared with others. And this even though in the highest risk group, the percentage of people who actually reoffend is only 30%.

It is in this aspect of relative risk that the most significant advances in criminological research on the risk of reoffending have taken place. When there are more than two possible levels of risk, the analysis of predictive accuracy can no longer only be limited to binary indicators such as sensitivity, specificity or predictive values, but other indicators have emerged that relate the multiple possible levels of risk to the outcome of recidivism or non-recidivism. The most commonly used indicator to assess the performance of structured risk assessment tools is the so-called area under the ROC curve,^31^ which measures relative risk: it says how likely it is that a randomly selected recidivist would have received a higher risk rating on the tool than a randomly selected non-recidivist (Singh, 2013). That is, it reports how well the tool discriminates between higher and lower risk individuals but says nothing about the reoffending probabilities associated with each level. For example, in one tool the low-risk group may have an associated reoffending probability of 5% and the high-risk group may have a reoffending probability of 15%, and an area under the ROC curve of 0.75 (which is conventionally considered a high value). In another tool the recidivism probabilities may be 10% and 50% respectively and have the same area under the ROC curve value of 0.75. In both cases there is a 75% chance that a randomly selected recidivist will have had a higher risk classification than a randomly selected non-recidivist. But being high risk is associated with a very different probability of reoffending in each of these instruments, and from the point of view of a judge seeking to impose a sentence commensurate with the risk level of the individual, it has a very different meaning whether the probability of reoffending associated with being high risk is 15 or 50%.^32^

On the other hand, the area under the ROC curve summarizes the discriminatory power of the risk assessment tool in a single number (e.g., 0.75), but the false positive and false negative rates can vary dramatically depending on which discrimination threshold is used to make a particular decision. For example, suppose we were to use a risk assessment tool that classifies people into five risk levels (very low, low, medium, high, very high) and has an area under the ROC curve of 0.75, to decide whether to grant parole. If we decide to parole only those who are classified as very low risk, we will have many false positives and very few false negatives; if we parole all but those in the very high risk group, we will have many more false negatives and fewer false positives. None of these error rates need to coincide with the area under the curve (75%), and both false positives and false negatives can be well above or well below 75% for each of our decisions, depending on the threshold we have set in each case.^33^

For these reasons, several authors have warned that the value of the area under the ROC curve alone is very uninformative when assessing the usefulness of a risk assessment tool for legal decision-making (Szmuckler et al., 2012; Shepherd and Sullivan, 2017; Fazel, 2019, p. 198), and recommend complementing the analysis of predictive accuracy with information on other statistical indicators, and always reporting the limitations of all of them (Singh, 2013; Rossegger et al., 2014; Douglas et al., 2017).

The predictive accuracy of risk assessment tools can indeed be expressed by many different statistical indicators, each of which measures a different dimension of this accuracy (Singh, 2013; Muñoz Vicente and López-Ossorio, 2016; Loinaz, 2017, p. 87 ff.). For example, the same tool may have a very high sensitivity but a low specificity, or an acceptable area under the ROC curve but a very low positive predictive value. This means that the predictive ability of a risk assessment tool can sometimes be described as both “good” and “bad,” if some of the indicators reach very satisfactory levels, while others remain at much more modest levels.

If this is the case, it is very important that the information provided on the predictive accuracy of these tools covers the various possible dimensions; otherwise, if the information includes only those indicators that yield higher values, and omits others that are less satisfactory, the impression is given that the overall performance of the tool is better than it actually is. However, it is very common for studies to report only relative risk indicators (and in particular the area under the ROC curve), explicitly stating that these achieve acceptable or satisfactory values, but not providing information on absolute risk, i.e., the probability of reoffending or violent reoffending associated with each level of risk, nor do they usually provide the positive and negative predictive values associated with each discrimination threshold.

Because of all these problems, and some others that risk assessment also presents,^34^ it has been recognized in criminology that “not only is the predictive accuracy of risk assessment tools imperfect, it is also imperfectly presented in the literature. This limited and skewed evidence base creates a risk that decision makers will rely more heavily on risk assessment scores than their accuracy warrants” (Douglas et al., 2017, p. 135).^35^

In short, notwithstanding the fact that risk estimates work much better for other things (detection of low-risk individuals, ability to discriminate within a group between individuals at higher risk than others), the empirical evidence accumulated over the last 40 years shows time and again that we are still wrong more than half the time when we make estimates of high risk of violent crime. And although it is sometimes criminologists themselves who explicitly warn of this problem and of the consequences of using these estimates as the basis for measures that severely restrict rights,^36^ advocates of evidence-based sentencing tend to ignore this when they appeal in a general way to the fact that structured estimates of risk of reoffending are “much better” than the old structured clinical assessment and should therefore be used in choosing the type of sanction and the way in which it is carried out.

### 3.2. Is there empirical evidence that evidence-based sentencing achieves its goals?

It is relatively common for evidence of the success of EBS to be cited, for example, that judges and other actors in the criminal justice system find the information provided by risk assessments useful, or that as a result of the introduction of EBS the number of prison sentences has been reduced and the number of alternative sentences increased.^37^ But while all these results are certainly positive, they do not provide direct information on the two main objectives of EBS, which are to reduce reoffending and the costs associated with the overuse of the prison system, and to control crime rates. As the evidence-based policy it claims to be, evidence-based sentencing should be able to demonstrate with empirical evidence that these outcomes are actually achieved; in other words, that “it works.”

However, whereas there are many studies on the predictive validity of risk assessment tools, far fewer have studied their usefulness in reducing recidivism or crime rates (Viljoen et al., 2018, p. 184). And those that do exist do not yield very encouraging results for EBS. As Stevenson puts it, “Somehow, criminal justice risk assessment has gained the near-universal reputation of being an evidence-based practice despite the fact that there is virtually no research showing that it has been effective” (Stevenson, 2018, p. 306).

A systematic review that included studies published up to 2017 concluded that there is insufficient empirical evidence to claim that the use of structured risk assessments reduces violence or reoffending, because the available studies, in addition to having a number of important methodological limitations, show mixed results: while in some cases a reduction in violence or crime rates is observed after the use of risk assessments, in others this is not the case (Viljoen et al., 2018, p. 200, 204). It is also worth noting that eight of the 12 studies analyzed in this review were conducted on samples of psychiatric patients (Viljoen et al., 2018, p. 198), which would pose significant problems in generalizing the results to the standard offender population, even if an association between the use of structured risk assessments and reductions in offending had been demonstrated.

In another systematic review of 22 studies involving 1,444,499 adolescents and adults, the authors found that the use of risk assessment tools was associated with a small overall reduction in restrictive placements, particularly for low-risk individuals, and a small reduction in any reoffending, but after removing studies with a high risk of bias, the results were no longer significant. They also concluded that much of the available research of poor quality and that there is a strong need for more rigorous research before clear conclusions can be drawn (Viljoen et al., 2019, p. 1, 401–411).

A thorough empirical evaluation of pretrial risk assessment in Kentucky found that the 2011 bail reform, which mandated the use of pretrial risk assessment with the explicit goal of lowering incarceration rates, did not achieve the intended effects although it did change bail-setting practices, and after a couple of years the pretrial release rate was lower than it was before the reform (Stevenson, 2018, p. 308–311).

Empirical research on the implementation of EBS in the state of Virginia has also been published (Stevenson and Doleac, 2019).^38^ Virginia was the first state in the US to systematically implement risk assessment in sentencing in 2003 for non-violent and sex offenders. Risk assessment was incorporated into the state's sentencing guidelines with the goals of: (a) providing alternatives to prison for a significant number of low-risk, non-violent offenders, and (b) allowing for longer sentences for high-risk sex offenders. And in both cases only as a recommendation to the judge, who decides whether or not to follow it. This policy was driven, as is characteristic of EBS, by considerations of cost-effectiveness: reserving expensive prison places for the most violent offenders while maintaining a high level of public safety.^39^

The research to which we refer analyses the impact of these changes on the prison population and reoffending rates, and the results are remarkable. On the one hand, for the group of non-violent offenders, neither the prison population rate nor the number of sentences imposed decreased. However, this does not mean that judges did not taken into account the recommendations derived from the risk levels. According to the study, there are differences in the likelihood of being sentenced to prison, and also in the length of the sentence, between non-violent offenders below and above the cut-off point that marks the boundary between high and low risk. What happened is that the reduction in the number and length of prison sentences for the low-risk group was offset by an increase in both factors for the high-risk group, so that the net effect of the implementation of these policies on the total number of prisoners ended up being zero (Stevenson and Doleac, 2019, p. 2, 3, 19).

One might have thought that even if the prison population had not been reduced, increasing the severity of sentences for the most dangerous offenders would at least have achieved the second objective of reducing reoffending. However, the data show that this is not the case either (Stevenson and Doleac, 2019, p. 2, 20): recidivism rates did not change significantly.

On the other hand, the results for the sex offender group are also striking: while for these cases the explicit purpose of the reform was to allow increases in sentence severity above the recommended guidelines only for high risk, the study shows that after the introduction of risk assessment there was a 5% decrease in the likelihood of being imprisoned and an ~24% decrease in sentence length (Stevenson and Doleac, 2019, p. 19).

The study suggests several explanations for these surprising results. A very important one is that the judges did not always follow the recommendations: in addition to the guidelines and risk assessments, they also took other criteria into account, among which age stands out. Being young is one of the most risk-aggravating factors in any assessment tool, including the one used in Virginia. However, the courts have traditionally viewed youth as a mitigating factor in determining liability. The study shows that judges in Virginia did modify sentences on this point to conform to the recommendations of the risk assessments: there was a relative increase in the severity of sentences imposed on young people, and also in the likelihood that they would receive a prison sentence. But the adjustment was only partial, because if judges had consistently followed the recommendations in all cases, these increases would have been multiplied (Stevenson and Doleac, 2019, p. 3, 4).

With regard to the sex offender group, the study suggests two possible explanations. One is that judges had a preconceived notion that this group of offenders was more likely to reoffend than they actually were, and thanks to the risk assessments they realized that they were less dangerous than they thought, which would explain the reduction in the severity of sentences. However, Stevenson and Doleac believe that another hypothesis is more plausible: that low-risk assessments have been used by judges as a “shield” to impose sentences that they consider more appropriate, but which they have previously been afraid to impose because of the huge costs to their prestige and professional careers that false negatives entail. A low-risk assessment would make it possible to shift some of the responsibility in the event of a repeat offense by someone who could have been imprisoned if he or she had received a longer sentence (p. 19, 20).

As for the fact that recidivism remained constant after the introduction of risk assessment, the authors of the study rule out as a possible explanation that the tool used in Virginia is flawed or poorly designed, and simply point to the fact that recidivism is a very difficult phenomenon to predict under any circumstances. In their view, risk estimates explain only a tiny percentage of recidivism, so that adopting one policy or another on the basis of these estimates can have only a very limited effect on the variation in reoffending rates (Stevenson and Doleac, 2019, p. 33 ff.).^40^

## 4. Discussion

So far, there is no scientific evidence that EBS achieves its intended goals of reducing reoffending and prison use. However, it may be that this ineffectiveness is not due to any inherent shortcomings of EBS, but to its misapplication in practice. The study by Stevenson and Doleac could point in this direction: since the risk recommendations in the Virginia case were not binding, judges did not always follow them; if they had, the results would have been better, so what needs to be done is to eliminate judicial discretion and make it mandatory always to tailor sanctions to what the risk levels recommend.

However, there is a widespread view among advocates of EBS that risk assessments should not be binding on judges.^41^ It is recognized that, in addition to the level of risk, there may be other important criteria to be taken into account in sentencing and corrections, such as the availability of resources to carry out the treatment or the type of supervision ordered. It is also generally accepted that the reduction of recidivism is only one of the various objectives pursued by the criminal law, so that considerations such as the seriousness of the offense or the need for general deterrence may legitimately lead to the imposition of sentences which are not commensurate with the risk of reoffending.^42^ As far as the academic field is concerned, most proponents of EBS place it within a framework of limited retributivism that sets maximum (and sometimes minimum) limits beyond which sanctions cannot be imposed, even though they may be appropriate according to the level of risk.^43^

This creates the following paradox: if it is necessary to adhere strictly to risk-based recommendations in order to achieve the intended benefits of EBS (in terms of reducing crime and reoffending), but at the same time there are good reasons for not doing so, which must be respected and which make it impossible, then it would seem that the proponents of the practice themselves are acknowledging the impossibility of its success.^44^

On the other hand, it is highly doubtful that even if sentencing were to be based solely and exclusively on risk level, the desired objectives could be achieved. This is partly because, as we have already seen, the scientific evidence on which evidence-based sentencing is based is much less robust than it might appear at first sight. The high rates of false positives raise serious questions about the efficiency of a system that would systematically devote excessive resources to intensive criminal control of people who do not need it. And the concern for reoffending that underpins the whole approach reflects an intolerance of false negatives that would probably also favor a penal response to low-risk groups that is disproportionate to the real risk they pose. Moreover, if it is not easy to estimate the risk of reoffending with a high degree of accuracy, it is even more difficult to reduce that risk. There are, of course, effective treatment programmes for reducing reoffending,^45^ but there does not appear to be conclusive empirical evidence that the use of risk assessment is successful in reducing reoffending. This may be for a number of reasons, including the very obvious one that risk assessment alone is unlikely to reduce risk unless it is followed by intervention,^46^ or, as Monahan and Skeem argue, that there is not yet enough good empirical research on which risk factors are causal and therefore which need to be modified to reduce the risk of reoffending.^47^ But in any case, an approach that claims to be “evidence-based” and that claims to reduce reoffending with risk assessments should, in my view, be able to provide data to support this claim.

What is more, risk assessments are not the only knowledge about crime that criminology has produced in its long history: on the contrary, there is scientific evidence that would support penal policies quite different from those advocated by EBS. Take age, for example: being young is one of the factors most directly correlated with a higher risk of reoffending and violent recidivism, and is therefore included in virtually all assessment tools. Age can explain almost 50% of the risk score in structured instruments, and its weight in the total score is often equal to or greater than that of criminal history.^48^ However, empirical evidence on criminal careers and age curves also shows that while the proportion of people who commit crimes in adolescence and early adulthood is very high, the vast majority of them stop a few years later, in their early twenties. And that, even within the group of those who can be considered career criminals, many drop out at a relatively early age (in their thirties).^49^ This being the case, imposing long sentences on young people, and even very long sentences on relatively young people with long criminal records, can have only a very limited incapacitating effect, since many of these individuals would have given up crime anyway, and therefore the considerable resources allocated to their imprisonment cannot be considered an efficient investment from a cost-benefit point of view. In other words, even if we remain within the utilitarian and efficiency-based logic of EBS, there is empirical evidence that seriously challenges the claim that imposing more intensive (and more expensive) penal control on those who, because of their age and criminal history, are at high risk of reoffending, is in fact an efficient investment.^50^

There is yet another consideration to be made in relation to age as a risk factor and scientific evidence. We have already mentioned, there is a well-established tradition in criminal law of valuing youth as a mitigating factor. Most countries have specific juvenile justice systems that operate based on re-education criteria and provide for comparatively lighter penalties than those imposed on adults who commit the same offense. This is justified by the fact that social sciences, and psychology in particular, show that minors, even if they know the rules from a certain age onwards, are generally still immature, highly impressionable, impulsive, and with a lower capacity to tolerate frustration. These psychological characteristics merit the application of milder punishments from a retributive perspective. More recently, neuroscience has confirmed that there are not only psychological differences between adolescents and adults, but that the degree of brain development differs between them, and that this difference persists not only until the legal age that marks the borderline for treatment as an adult in many countries (around 18 years), but also until the early twenties.^51^

There is, therefore, empirical evidence to justify imposing more intensive penal regimes on young adults because of the greater risk of reoffending they represent, if we consider that incapacitation is the central aim of the penal system. And there is also empirical evidence to justify a more lenient penal response for the same group of people if we consider that punishment proportionate to the degree of responsibility should be the central criterion for sentencing. Much the same can be said for other factors, such as certain mental illnesses.^52^ In other words, depending on which goals we consider to be a priority, we have the scientific evidence to design very different criminal justice policies, all of which would be equally evidence-based.

In the design of criminal policy, scientific evidence is sometimes important and sometimes not. There are areas that have proved to be impervious to the social science evidence accumulated over the years on the zero or very limited effectiveness and the enormous side effects of certain practices (for example, the death penalty or the “war on drugs”), and others where scientific evidence has been more or less successful in penetrating (for example, policing and some areas of rehabilitative penal enforcement). The main reasons why criminological evidence is or is not taken into account are not primarily related to its scientific quality, but to other factors: whether or not it fits in with the political objectives pursued by governments at a given time, whether it coincides with a window of opportunity to be well received by public opinion, whether or not there is pressure from certain interest groups for or against the inclusion of this evidence, and so on (Tonry, 2013a). The same is true of evidence-based sentencing: there is no more or better scientific evidence to support it than there is to support other models, and the success of this approach in the US is due to other factors.^53^

Consequently, the term “evidence-based” (which implies that other policies would not be based on science, or would have less scientific support) is not justified in my view, and it would be preferable to refer to this approach by one of the other terms that, as we saw at the beginning of this paper, describe its content well: predictive sentencing or risk-based sentencing, for example.

What is more, evidence-based sentencing suffers from some of the problems that plague “evidence-based policy” described in section 2.3 of this paper. It presents itself as based on “the best available evidence” but fails to make explicit the many shortcomings (as well as the undoubted virtues) of structured risk assessment for reoffending, and thus offers at best an incomplete (and at worst a biased) picture of the true state of scientific knowledge about the predictive capacity of risk assessment and its actual impact (or lack of impact) on crime rates. On the other hand, it encourages (either deliberately or inadvertently) a depoliticisation of the criminal justice debate by presenting risk-based sentencing as a practice that “works,” and should therefore be accepted, when the crucial question is: it works for what? Evidence-based sentencing assumes that the “what for” must be the reduction of reoffending, but it has not yet been shown to do so, nor is it at all clear that this should be the primary aim of the criminal justice system. There are many other possible and legitimate objectives, such as ensuring the non-discriminatory application of sentences, promoting the rehabilitation of as many offenders as possible, avoiding the imposition of disproportionate sentences, or, of course, reducing crime rates, which is not the same as reducing reoffending rates. The debate about which of these goals should be preferable or a priority necessarily involves value-based arguments (normative, political, ethical), and although it can (and must! if it is to be rational) also deal with arguments related to what criminology knows about the effects of punishment, it cannot be replaced by them.

This is why I believe that evidence-based sentencing, at least in the form that it has taken in recent years in the US, is not a truly evidence-based practice, but rather an example of the selection of certain scientific evidence to justify certain public policies (policy-based evidence), hiding behind supposedly technical reasons options that can only be the subject of political debate.

Finally, I would like to stress that the criticism leveled at EBS in no way detracts from the fact that various rehabilitation programmes have shown good results in reducing reoffending. I also believe that risk assessment tools do a reasonably good job of discriminating between groups at higher and lower relative risk, and that they identify the lower-risk groups with a remarkable degree of accuracy, which can help with allocation to treatment programmes and provide a strong argument for widening the range of alternatives to prison. Effective rehabilitation can (and in my view should) have an important place in the enforcement of sanctions. But this does not mean that there is a sufficient scientific basis—nor, in my view, better reasons—to make so-called “evidence-based” sentencing the cornerstone of the criminal justice system.

## Author contributions

LM-G: Writing—original draft, Writing—review & editing.

## Footnotes

^1^This has not been the case in other aspects of US crime policy, which have been much more receptive to the knowledge provided by criminology (Tonry, 2010).

^2^Data taken from Bureau of Justice Statistics, Key Statistics, “Estimated number of inmates held in local jails or under the jurisdiction of state or federal prisons and incarcerationrate, 1980–2016” at http://www.bjs.gov (last visited 22.08.2020). The rate amounts to 1,000/100,000 if calculated with respect to the total adult population residing in the USA.

^3^National Research Council, 2014. A summary of the main indicators up to 2017 can be found in The Sentencing Project, Criminal Justice Facts, “Fact sheet: Trends in U.S. Corrections,” at https://www.sentencingproject.org/criminal-justice-facts/ (last visited 22.08.2020).

^4^It is common to explain the abandonment of indeterminate sentences from the late 1970s onwards as a transition toward a model inspired by retributive ideals. Although this description does fit with the reforms that took place until the mid-1980s, for those produced since then it is difficult to identify the fundamental logic of any retributive paradigm: proportionality with the seriousness of the offense committed (Tonry, 2013b, p. 145, 150).

^5^Cf. also the definition provided by Burke in the *Science Bench Book for Judges*: “The goal of evidence-based sentencing is […] to more effectively: identify who may be safely and effectively supervised in the community; and, order appropriate conditions of community supervision given the defendant's recidivism risk, criminogenic needs, and responsivity factors” (Burke, 2020, p. 279–280).

^6^“The principal use of EBS practices at the state trial court level is the use of actuarial risk- and needs-assessment information in state sentencing proceedings” (Warren, 2010, p. 156).

^7^As Marcus states: “The resulting critical issue in sentencing policy thus runs along a very different axis than the traditional divide between punitivists and advocates of reformation. Evidence-based ‘smart sentencing' posits that by rigorously scrutinizing data on what works or not on which offenders, we can allocate our correctional resources far more efficiently—measured by public safety—than if we continue to settle for ‘just deserts' with no accountability for outcomes” (Marcus, 2006, p. 57). In a very similar vein, cf. *Principles of Effective State Sentencing and Corrections Policy*, published by the National Conference of State Legislatures (2011, section “Modern Sentencing Strategies”): “Many of these approaches leave behind outdated notions of being ‘soft' or ‘tough' on crime. Instead they look to be smart on crime to ensure that sentencing policies contribute to a favorable state return on public safety expenditures.”

^8^In an updated list as of 2019, Stevenson and Doleac list 28 states, and seven others in which a county also uses it (Stevenson and Doleac, 2019, p. 54–55).

^9^Cf., for example, the strongly critical position of Starr, 2014, p. 803–872. In Spanish, an overview of the state of the issue can be found in Martínez Garay, 2019, p. 149 et seq. A recent and highly critical contribution to both the use of risk assessments and neuroscientific knowledge in criminal proceedings is that of Julià Pijoan, 2020 (with specific references to evidence-based sentencing on p. 279 et seq.).

^10^Cullen clearly describes the strong opposition that rehabilitation advocates faced in the 1980s and 1990s, and the efforts they made to provide “numbers” to convince skeptics that there was evidence that certain interventions worked (Cullen, 2013, pp. 335 ff.).

^11^An expression which became popular after Robert Martinson's well-known work published in 1974: “What works? Questions and answers about prison reform,” *The Public Interest*, 35, p. 22–54.

^12^For example, *Public Safety Policy Brief* No. 8 (May 2009) edited by the PEW Center on The States as part of its Public Safety Performance Project, entitled “Arming the Courts with Research,” states that “we cannot afford to ignore the opportunity to reduce offender recidivism and resulting high crime rates through use of these cost-effective evidence-based practices,” and on the following page qualifies: “The goal of recidivism reduction is to reduce crime, not just to rehabilitate offenders [...]. It is not 'soft' on crime” (accessible online at: https://cdpsdocs.state.co.us/ccjj/Resources/Ref/2009-05_PEW_ArmingTheCourtWithResearch.pdf, last accessed 25.09.2023). The Guide published in 2011 by the National Center for State Courts on EBS emphasizes that the primary goal is to “reduce and manage the risk” posed by the offender in order to better protect public safety. The few references to rehabilitation in the guide place it on the same level as other legitimate purposes of punishment such as incapacitation, or specific deterrence (Casey et al., 2011, p. 1, 11, 12).

^13^The National Center for State Courts is one of the few institutions that expressly stated not to pursue such an objective (see “NCSC Fact Sheet on Evidence-Based Sentencing,” 2014, accessible online at https://cdm16501.contentdm.oclc.org/digital/collection/criminal/id/475, last visited 25.09.2023). However, a more recent document from the same organization acknowledges that risk assessments can be a contributing factor to avoid imposing prison sentences on low-risk offenders (Casey et al., 2017, p. 3).

^14^Monahan and Skeem put it clearly when they ask themselves why, after 40 years of “just deserts,” we are witnessing a remarkable resurgence of risk as an essential component in sentencing and enforcement: “Money appears to be the principal answer” (Monahan and Skeem, 2014, p. 158).

^15^The PEW Center's *Public Safety Policy Brief* referred to above (at note 16) puts it very clearly: “The goal of recidivism reduction is to reduce crime, not just to rehabilitate offenders. It includes both effective treatment services—programs proven to reduce reoffending—and swift and effective use of graduated sanctions. It highlights the importance of holding offenders more strictly accountable than we do now for compliance with courtorders and conditions of supervision. It is not ‘soft' on crime.”

^16^For an overview of the background and emergence of this trend, see Parkhurst, 2016, p. 14 ff.

^17^One such argument can be found in the document “Principles of Evidence-Based Policymaking,” produced by the Evidence-Based Policymaking Collaborative (comprising various foundations and organizations such as the Laura and John Arnold Foundation, the Urban Institute and the Brookings Institution): “In an era of intense partisanship and constrained public resources, evidence-based policymaking can help bridge the partisan political divide and support research-based debate about what outcomes we want to achieve, for whom, and at what cost. It encourages transparency and accountability [...] (p. 1 f.)” (accessed online at https://www.urban.org/sites/default/files/publication/99739/principles_of_evidence-based_policymaking.pdf, last accessed 25.09.2023).

^18^“Just as no-one would argue that clinicians should practice medicine without regard to evidence, so it would seem an incontestable, self-evident proposition that policymakers should base their decisions on evidence” (Klein, 2000, p. 65).

^19^In relation to criminal law it has been argued that “the evidence-based revolution in medicine provides a model for how a tradition-bound profession can change its approach to supplement and inform traditional discretion with sound scientific research” (Garrett, 2018, p. 1496).

^20^Among the most noteworthy was that provided by Tony Blair's British Labor government, which in the 1990s explicitly incorporated the evidence-based policy approach into its programme with the declared aim of “removing ideological and partisan conceptions from the political decision-making process, grounding these decisions in evidence based on scientific research [...] about what worked and how better results could be produced” (Benito Sánchez, 2020, p. 28). This same example, however, also serves to illustrate some of the problems I will tackle in the text: in 1999, the British Labor government launched the *Crime Reduction Programme*, which aimed to obtain solid scientific evidence on which strategies “work” in crime control and which do not, and then transfer this knowledge to the design of crime policies. This programme, however, unfortunately failed shortly afterwards and was canceled without having achieved almost any of its objectives. On the characteristics of this programme and the difficulties it faced, see Hope, 2004; Hough, 2004; Maguire, 2004. The “what works” agenda in the UK has been revived since 2013 under the “What Works for Crime Reduction Center” in the UK College of Policing, that with a much more modest budget collects and shares research evidence on crime reduction and support its use in practice, including a practitioner toolkit. As Tilley and Laycock (2018) explain the Center has been moving toward a realist evaluation approach and away from a focus on just randomized control trials and meta-analyses.

^21^Even in the very field from which evidence-based practices emerged: medicine. For an assessment of the current state of evidence-based medicine and the problems it faces, see Ioannidis, 2016, 2017, and the various papers published in the monographic issue of the *Journal of Clinical Epidemiology* 84 (2017).

^22^The very determination of what are the facts, of what is to be considered as “evidence” and what is not, is influenced by the relative power and influence that different epistemological traditions have in each country, culture, and historical moment (Strassheim and Kettunen, 2014, p. 260). This consideration is particularly relevant for the purpose of this paper, which analyses the existing empirical evidence on risk assessments (cf. *infra*, section 3.1): precisely the concept of “risk” is far from referring to an objective reality external to the observer because (with the exception of strictly realist theses) risk is what which *is perceived* as dangerous in a given historical and geographical context, and is therefore mediated by cultural and social processes; on the notion of risk, cf, Brandariz García, 2016, p. 50 y ss.; Castro Liñares, 2019, pp. 33 ff.

^23^Hammersley, 2005, p. 94 et seq.; on the simplification associated with “what works” analyses and the dangers it entails, see in detail Parkhurst, 2016, p. 18 et seq.

^24^Greenhalgh and Russell, 2009, p. 307; Hammersley, 2005, p. 89 ff. As Klein argues, in the case of social science, research tends to raise as many new questions as it solves (Klein, 2000, p. 65).

^25^And, of course, in the accumulated knowledge of rehabilitation programmes “that work.” However, as we have already noted above, the central premise of the EBS is that sentencing must be adapted to the level of risk of each individual, not only in order to apply rehabilitation programmes, but also purely incapacitation programmes in cases where the latter are more effective. And in both cases, the necessary condition is that it is possible to estimate the risk with a sufficient degree of accuracy: if this were not possible, there would be no logic in designing sentencing and corrections with a view to the risk and needs of each individual.

^26^On the different risk assessment techniques and their characteristics, see Loinaz, 2017.

^27^Cf. e.g. Ægisdóttir et al., 2006.

^28^Fazel et al., 2016, p. 539, 540: 20% in predicting violent recidivism in a one-year period; 37% if the period extends to 2 years. Similar Fazel et al., 2019, p. 4: 17% for violent recidivism at one year; 20% at 2 years.

^29^If, instead of the commission of new violent crimes, the risk of committing any type of crime is estimated, the positive predictive values are higher: 52% on average in the meta-analysis by Fazel et al., 2012, p. 10; between 40 and 60% in the study by Fazel et al., 2019, p. 4 (depending on the characteristics of the sample and the risk level at which the discrimination threshold is set); or even 75% in the study by Fazel et al., 2016, p. 540. The problem is that this value is much less relevant from a criminal justice point of view, as it is highly questionable whether the likelihood of committing non-violent and low-level crimes justifies (neither from a purely economic rationale nor from a value-based perspective) different regimes of severity of punishment and intensity of supervision.

^30^And even this is a remarkable oversimplification of the problem, for it makes no difference whether a new offense of theft or murder is committed, whether only one new offense is committed or many, whether this recidivism is observed a few months after release from prison or many years later, and so on. If structured violence risk assessments cannot provide the judge with differentiated information on these points, it is doubtful whether they can be considered fit for purpose from the perspective of the criminal justice system (Slobogin, 2019).

^31^ROC for *Receiver Operating Characteristics*.

^32^On the meaning and information provided by the area under the ROC curve see, in more detail, Mossman, 1994, and Martínez Garay and Montes Suay, 2018.

^33^For an example of how the area under the curve can “mask” very large error rates in risk assessment, depending on the cut-off point chosen and the prevalence of the phenomenon, see Hester (2019, p. 220–226) (recounting the actual case of the design of a risk assessment tool in Pennsylvania).

^34^For example, conflicts of interest that go unrecognized and signs of publication and authorship bias (Douglas et al., 2017, p. 135; Fazel, 2019, p. 202); the high complexity of statistical indicators is such that researchers themselves sometimes use them incorrectly (Singh et al., 2013); many tools have not been externally validated (Douglas et al., 2017, p. 153) and when validation studies exist, they do not always respect the essential characteristics of the original study (Rossegger et al., 2013); recidivism rates for each risk level vary markedly between samples (Rossegger et al., 2014; Singh et al., 2014); when risk assessments are applied in everyday criminal justice practice, the levels of accuracy are lower than those found in academic studies (Fazel, 2019, p. 203).

^35^An even harsher judgement can be found in Fazel, 2019, who, after listing a series of scientific validity criteria that risk assessment tools for recidivism or violence should meet, states that very few of the currently existing tools meet more than one or two of them, and concludes that “the most commonly used tools in criminal justice are not suitable for predictive purposes” (p. 207).

^36^Cf. e.g., Yang et al., 2010, p. 761, stating that due to their moderate levels of predictive efficacy, risk assessment tools should not be used as the sole or primary means of making clinical or judicial decisions that depend on a high level of predictive accuracy, such as preventive detention. Cf. also Fazel, 2019, p. 199, 201.

^37^E.g., Casey et al., 2017, p. 4. In this vein, Ostrom and Kauder (2013) positively assess the introduction of EBS in the State of Virginia (to which we will refer in more detail below), offering data on the level of follow-up of recommendations by judges, on how much the workload of supervisory agents increases, or on the degree of satisfaction expressed by various actors in the system.

^38^Another experiment in the application, not really of EBS but rather of evidence-based corrections on a large scale, is the *Justice Reinvestment Initiative*, to which we cannot discuss in more detail in this paper for reasons of space, cf. Klingele, 2015, p. 562 ff; Garrett, 2018, p. 1503 ff; Austin et al., 2013 (passim) as well as Sabol and Baumann, 2020, passim.

^39^Cf. Ostrom and Kauder, 2013, p. 161, explaining the evolution of offender risk assessment in Virginia: “Viable strategies are needed that allow states to reserve scarce and expensive prison beds for the most dangerous offenders and use less costly punishment options for less serious offenders—all while maintaining public safety.”

^40^The study only analyses the evolution of recidivism rates in the group of low-risk offenders; it does not analyse recidivism rates in the group of sex offenders due to the lack of available data, as this is a small group with very long sentences that were still being served at the time of the analysis.

^41^Cf. National Center for State Courts, *NCSC Fact Sheet. Evidence-Based Sentencing* (2014): “EBS does not replace judicial discretion. EBS provides additional information for the judge to consider in crafting an offender's sentence. Judges are free to use the information as they deem best in light of all the facts of the case and the parties' presentations in court to address all the purposes of sentencing” (available online at https://cdm16501.contentdm.oclc.org/digital/collection/criminal/id/475, last accessed 25-9-2023).

^42^Casey et al., 2011, p. 11–14.

^43^E.g., Monahan and Skeem, 2014, p. 158; Slobogin, 2019, p. 108 ff.

^44^In my opinion, Slobogin comes to a similar conclusion (Slobogin, 2018, p. 587, 593), although he insists on defending what he calls risk-based sentencing, mainly because he believes that the alternative (the retributive or desert-based approach to sentencing) raises problems of equal or greater magnitude (Slobogin, 2019, p. 116 et seq.).

^45^The research accumulated since the 1970s has repeatedly pointed this out, highlighting as particularly effective those with a therapeutic orientation, focusing on cognitive skills training, drug treatment and educational programmes, while there is also evidence of what has not been shown to reduce reoffending: long prison sentences, militarized regimes of incarceration such as *boot camps*, or intensive supervision in the community; cf, for example, MacKenzie and Farrington, 2015.

^46^This is known as “risk management”, which is a key issue, and more complicated than mere risk assessment, because it depends not only on whether there are programmes in place, but also on whether there are sufficient resources to implement them, and the capacity and willingness to do so rigorously. Otherwise, mere risk assessment is likely to be a waste of time (Monahan and Skeem, 2014, p. 162 f.); Viljoen et al. (2018, p. 182, 204) add that according to some authors it could even be considered harmful and unethical.

^47^Monahan and Skeem, 2016, p. 497 ff, 506. Cf. also MacKenzie and Farrington, according to whom, while we can “draw some conclusions about which general categories of interventions are effective, we are still far from being able to identify the specific intervention components that lead to desirable changes in particular types of offenders” (MacKenzie and Farrington, 2015, p. 590).

^48^Cf. Stevenson and Slobogin, 2018, p. 694: in the COMPAS tool, which is widely used by different jurisdictions in the US, age explains 48% of the variation in risk scores. The authors point out that they did not have access to the details of how the algorithm that runs COMPAS works, as it is proprietary, and that the calculations are therefore approximate (see Stevenson and Slobogin, p. 690 ff., 695 ff.). The relationship between the weighting of age and criminal record in different risk assessment tools can be seen in in the same paper, in a table at p. 699.

^49^Cf. Tonry, 2013b, p. 182, with further references.

^50^Tonry, 2013b, p. 182; Brandariz García, 2016, p. 236.

^51^Sánchez Vilanova, 2017, p. 208 et seq; Pozuelo Pérez, 2015, p. 5–10; O'Rourke et al., 2020, p. 5 et seq, p. 56.

^52^Monahan and Skeem, 2016, p. 504 f. give the example of the trauma of war veterans.

^53^Among these are that its promises of cost-effectiveness are welcome in times of economic crisis; that its emphasis on reducing reoffending can bring together politically distant actors (some share the goal of providing alternatives to prison for more people, while at the other end of the ideological spectrum there is a shared goal of keeping the most dangerous offenders locked up for longer); its technocratic and actuarial language fits well with the managerial model currently dominant in US policy; its focus on the sentencing and enforcement phase means that it can be implemented without challenging ideologically thorny issues such as reducing sentences for serious crimes; it has been proposed at a time when crime rates are very low, etc.
